# Redefining cardiometabolic biomarkers in the big data era: toward a personalized medicine-centered reconstruction of risk prediction models

**DOI:** 10.3389/fcvm.2026.1846105

**Published:** 2026-07-28

**Authors:** Xin-zhe Jiang, Li-jiao Guo, Ming-tian Zhang, Guang-ling Ji, Hong-tao Liu, Yue Zheng, Jie Zhou

**Affiliations:** 1 Department of Clinical Laboratory, The Affiliated Hospital of Changchun University of Chinese Medicine, Changchun, China; 2 Department of Gastroenterology and Digestive Endoscopy Center, The Second Hospital of Jilin University, Changchun, China; 3 Administrative Office, The First Clinical Hospital of Jilin Provincial Academy of Chinese Medicine Sciences, Changchun, China; 4 Department of Geriatrics, The First Hospital of Jilin University, Changchun, China; 5 Department of Cardiology, The Affiliated Hospital of Changchun University of Chinese Medicine, Changchun, China

**Keywords:** artificial intelligence, cardiometabolic biomarkers, causal inference, digital twin, multi-omics integration, precision medicine, risk prediction models

## Abstract

Conventional cardiometabolic risk prediction models rely primarily on population-derived averages and static biomarker thresholds, which inadequately capture individual biological heterogeneity and the dynamic nature of disease progression. Recent advances in multi-omics technologies, wearable sensing, and electronic health records have created new opportunities to move beyond static risk assessment toward individualized and longitudinal disease characterization. In this perspective article, we argue that cardiometabolic biomarkers should be redefined from isolated diagnostic indicators into dynamic biological anchors that reflect temporal trajectories, network-level biological states, and evolving responses to intervention. We propose an integrated translational framework that combines longitudinal biomarker monitoring, multimodal data integration, causal inference, personalized reference intervals, and adaptive risk prediction within a continuously learning precision medicine ecosystem. Within this framework, digital twin models serve as computational representations of individual biological states, enabling dynamic risk assessment and hypothesis generation for personalized intervention strategies.We further discuss key challenges that should be addressed before clinical implementation, including multimodal data harmonization, missing-data management, model interpretability, external validation, algorithmic fairness, data governance, and regulatory oversight. Finally, we outline practical priorities for future development, including longitudinal biomarker repositories, interoperable data infrastructures, diverse validation cohorts, clinician-facing decision-support systems, and prospective evaluation of adaptive biomarker-guided interventions. This perspective article reframes cardiometabolic biomarkers as dynamic components of individualized disease monitoring and decision-making systems, providing a conceptual roadmap for the next generation of precision cardiovascular medicine.

## 1 Introduction

### 1.1 Limitations of conventional models

Current cardiovascular risk prediction tools, such as SCORE2 and the Pooled Cohort Equations, are primarily based on traditional biomarkers, including blood lipids, glucose, and blood pressure (1, 2). These models are derived from cohort studies and apply population-level statistical associations to estimate individual risk (3). In doing so, they assume that group-derived averages can adequately represent individual pathophysiology. However, this approach overlooks the substantial biological heterogeneity among individuals (4). For instance, some individuals classified as having “metabolically healthy” obesity still experience cardiovascular events, while others develop adverse outcomes despite achieving target levels of conventional risk factors (5). This phenomenon of residual risk highlights the limitations of population-average models in capturing the complexity and dynamic nature of individual biological states, revealing a fundamental gap between population-based statistics and individual-level biology (3).

### 1.2 Opportunities afforded by big data

Advances in multimodal biological profiling and digital health technologies now provide unprecedented opportunities for individualised cardiometabolic risk assessment (6, 7). At the same time, wearable devices allow continuous, longitudinal monitoring of physiological parameters such as heart rate, physical activity, and sleep patterns (8). Electronic health records complement these data by providing comprehensive clinical phenotypes and treatment response trajectories over time (9). The integration of these diverse data sources creates a technological foundation for developing a new class of biomarkers that move beyond static thresholds to capture dynamic, real-time physiological states at the individual level (10).

### 1.3 Perspective of this article

**Figure 1:**
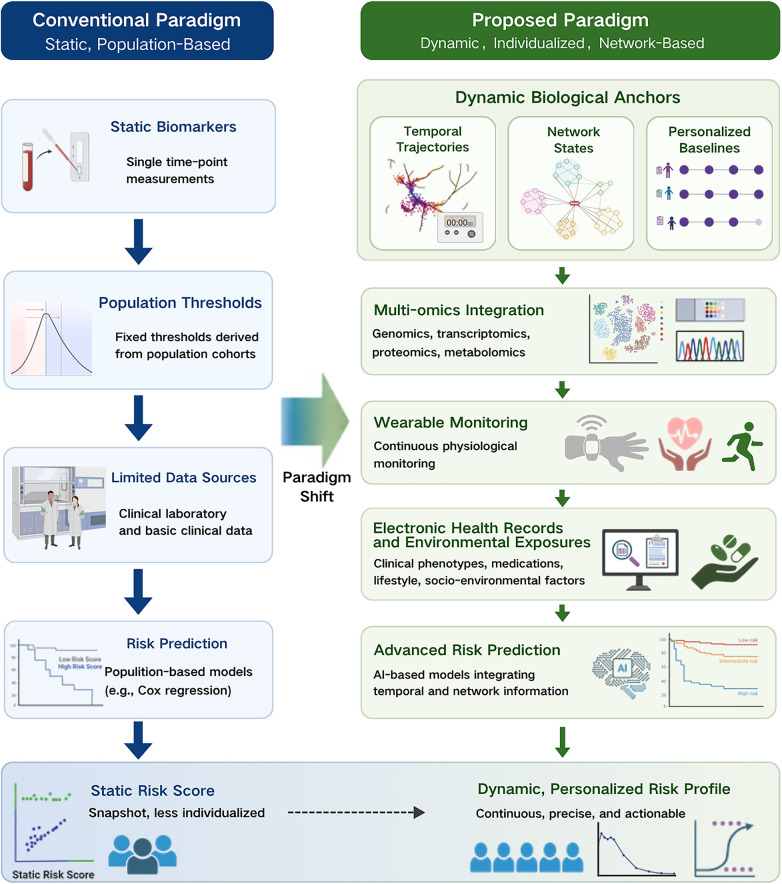
Paradigm shift of cardiometabolic biomarkers from static thresholds to dynamic networK,based systems. Traditional biomarker assessment relies on single time-point measurements and population-derived thresholds, which may overlook biological heterogeneity and temporal variability. The proposed framework redefines biomarkers as dynamic biological anchors that capture individualized trajectories and network-level biological states. By integrating longitudinal monitoring, multi-omics data, environmental exposures, and electronic health records, biomarker evaluation envolves from isolated measurements towards personalized, dynamic risk assessment, providing a more comprehensive representation of cardiometabolic pathophysiology. The figure was created using BioRender.com.

This perspective article argues that the definition of cardiometabolic biomarkers should undergo a fundamental shift: from serving as single-threshold diagnostic tools to acting as dynamic biological anchors that capture individualized trajectories over time. Dynamic individual trajectories refer to interpreting biomarkers within their temporal context and the internal environment of the individual, emphasizing patterns of change, rhythmic characteristics, and their integration within multi-omic networks—rather than relying on binary classifications based on fixed cutoff values. Based on this conceptual reorientation, a new generation of nonlinear risk prediction models can be developed that incorporate temporal information and individual biological backgrounds (Figure 1 and Table 1).

**Table 1:** Conventional versus personalized cardiometabolic biomarker paradigms.

Dimension	Conventional paradigm	Personalized paradigm
Data source	Single analyte	Multi-omics + wearable + EHR
Temporal assessment	Single time point	Longitudinal continuous monitoring
Biological representation	Static value	Dynamic trajectory
Data structure	Low dimensional	Multimodal high-dimensional
Model architecture	Linear regression/Cox models	Deep learning and digital twins
Risk interpretation	Population average	Individual-specific
Causal assessment	Association-based	Causal inference-guided
Clinical output	Static risk estimate	Dynamic risk dashboard
Clinical application	Episodic treatment	Closed-loop adaptive therapy
Main limitation	Biological heterogeneity ignored	Data integration and validation challenges

EHR, electronic health record.

Importantly, the novelty of this perspective does not reside in any single emerging technology. Rather, we propose a translational framework that integrates dynamic biomarker trajectories, multimodal biological networks, causal inference, and adaptive clinical feedback into a unified precision cardiometabolic ecosystem. Within this framework, cardiometabolic biomarkers evolve from static diagnostic indicators into dynamic biological anchors that continuously inform individualised risk prediction and therapeutic decision-making (11). Unlike earlier perspectives that primarily discuss digital twins, artificial intelligence, or multi-omics as independent technological advances, our framework emphasizes their integration within a coherent translational workflow that spans biomarker discovery, biological interpretation, causal inference, adaptive risk prediction, and clinician-oriented decision support (12–14). By focusing on systems-level coordination rather than isolated technological applications, this Perspective provides a conceptual framework for translating heterogeneous biological and clinical data into continuously updated, biologically informed, and clinically actionable risk assessment.

We contend that the future of cardiometabolic biomarker science should be defined not by the discovery of increasingly numerous biomarkers, but by the ability to contextualise biomarker information within individualised biological trajectories and actionable clinical decision pathways. From this perspective, the central challenge is no longer biomarker identification alone. Rather, it lies in constructing integrated systems capable of translating biological complexity into clinically meaningful intervention strategies. Accordingly, the principal contribution of this perspective article is not the introduction of a new analytical technology, but the establishment of an integrative conceptual framework that connects biomarker discovery, mechanistic understanding, dynamic prediction, and adaptive clinical implementation within a continuously learning precision cardiometabolic ecosystem.

## 2 New dimensions of biomarkers driven by big data

### 2.1 Beyond static thresholds: introducing the temporal dimension

Traditional approaches to biomarker-based risk assessment have relied heavily on single-time-point measurements, such as a one-time assessment of low-density lipoprotein cholesterol (15). Although convenient and standardized, such measurements offer an incomplete view of an individual's physiological state (16). A single measurement assumes that a biomarker's value remains stable over time—an assumption that often fails given the dynamic nature of biological systems (17). Variability in biomarker levels across repeated measurements, disruptions in circadian rhythms, and the cumulative burden of prolonged exposure to risk factors are all important dimensions of pathophysiology that a single assessment cannot capture (17, 18).

The concept of the exposome—defined as the totality of environmental exposures an individual encounters over the life course—provides a useful framework for integrating these temporal dimensions (19). By leveraging longitudinal data from electronic health records and continuous monitoring through wearable devices, it becomes possible to characterize not only the average level of a biomarker but also its visit-to-visit variability, circadian patterns, and cumulative exposure over time (20, 21). These temporal features enable a more nuanced understanding of risk. For example, high variability in blood pressure or lipid levels across repeated measurements has been shown to predict residual cardiovascular risk independent of mean values (22, 23). Incorporating such time-sensitive metrics into risk assessment moves beyond the limitations of static thresholds and aligns biomarker interpretation with the inherently dynamic nature of human physiology (24, 25) (Figure 1).

### 2.2 Beyond single markers: a network-based definition via multi-omics

The traditional paradigm of biomarker discovery has often followed a “one-marker, one-disease” logic, seeking to identify individual molecules that correlate with disease presence or outcome (26). Although this approach has produced clinically useful tools, it oversimplifies the complex biological mechanisms underlying cardiometabolic disease. Pathophysiological states rarely arise from changes in a single molecular entity; instead, they emerge from dysregulation across interconnected networks involving genetic susceptibility, protein signaling, and metabolic pathways (27).

Advances in multi-omics technologies enable a fundamental shift toward a network-based definition of biomarkers (28). Rather than focusing on isolated markers, this approach conceptualizes biomarkers as nodes within broader biological networks. Through clustering analysis that integrates genomics—such as polygenic risk scores—proteomics—including proteins like PCSK9 and inflammatory mediators—and metabolomics—such as branched-chain amino acids—it becomes possible to identify distinct cardiometabolic subtypes (27, 29). These subtypes reflect specific patterns of network-level dysregulation rather than elevations in any single molecule (27). In this framework, the true “biomarker” is not an individual analyte but the configuration of a perturbed regulatory network.

Several cardiometabolic conditions illustrate the potential utility of this framework. In patients with type 2 diabetes, integrating glycaemic variability, inflammatory biomarkers, and wearable-derived activity profiles may improve the prediction of cardiovascular complications beyond glycated haemoglobin alone (30). In individuals with apparently well-controlled low-density lipoprotein cholesterol, persistent elevations of interleukin-6 or high-sensitivity C-reactive protein may indicate residual inflammatory risk and identify candidates for anti-inflammatory interventions (31). Similarly, obesity-associated cardiovascular risk may vary substantially between metabolically healthy and metabolically unhealthy phenotypes, even among individuals with similar body mass index values (32, 33). Thus, network-based biomarker profiling may provide a more biologically informative classification than conventional risk factors alone.

## 3 Reconstructing risk prediction models: from linear regression to dynamic mapping

### 3.1 Upgrading model architecture

Conventional approaches to cardiovascular risk prediction have long relied on proportional hazards models such as Cox regression 34. While these models have proven useful in epidemiological research, they face inherent limitations when applied to the complex data landscape of modern precision medicine (34). Such models are not well suited for handling high-dimensional, sparse, or nonlinear data structures (35) (Table 2). They also struggle to integrate heterogeneous data types—including imaging, genomics, and unstructured clinical text—within a unified analytical framework (35, 36). Moreover, they typically assume proportional hazards over time and linear relationships between predictors and outcomes, assumptions that often fail to capture the true complexity of disease progression (36, 37) (Table 2).

**Table 2:** Comparison of conventional statistical models and next-generation AI-based prediction architectures for cardiometabolic risk prediction.

Dimension	Conventional Statistical Models	Next-Generation AI-Based Models
Statistical assumptions	Proportional hazards, linearity	Flexible nonlinear learning
Feature dimensionality	Limited	High-dimensional
Multimodal integration	Limited	Native multimodal fusion
Temporal modelling	Baseline measurements	Longitudinal dynamic learning
Nonlinear interactions	Pre-specified	Automatically learned
Missing-data handling	Conventional imputation	Representation learning/multimodal fusion
Model updating	Static	Continual learning
Interpretability	Regression coefficients	Explainable artificial intelligence
Validation strategy	Internal validation	External validation, calibration, transportability
Clinical output	One-time risk estimate	Dynamic individualized risk trajectory

AI, artificial intelligence.

The emergence of deep learning architectures, particularly Transformer-based models, offers a promising alternative. These models enable the integration of complex multimodal information and facilitate individualized risk representation (38). By processing sequential measurements, imaging features, genomic profiles, and clinical narratives together, such architectures can generate individualized risk maps that reflect the dynamic interplay among multiple biological and clinical factors (38, 39). This approach enables the creation of a “digital twin” for each patient—a computational model that evolves in parallel with the individual's physiological state (36, 39). In this context, a cardiovascular digital twin refers to a continuously updated computational representation of an individual's biological and clinical state (40, 41). Such a model may integrate multi-omic profiles, wearable-derived physiological signals, imaging findings, treatment history, and electronic health records (41, 42). As new information becomes available, the digital twin is iteratively updated to generate individualised risk projections, simulate potential responses to interventions, and identify emerging disease trajectories (41–43). Importantly, the validity of a digital twin should ultimately be assessed through prospective comparisons between predicted and observed patient outcomes (43).

Nevertheless, current evidence suggests that the performance gains achieved by advanced machine-learning and multi-omic models over well-established conventional risk prediction tools are often incremental rather than transformative (44, 45). Although these approaches frequently improve the integration of heterogeneous biological information, their predictive performance may decline when evaluated across independent external cohorts because of differences in patient populations, data quality, clinical practice patterns, and measurement protocols (44–46). Consequently, improvements in discrimination should be interpreted together with complementary measures of model robustness, including calibration, transportability, and demonstrated clinical utility, rather than being considered sufficient evidence for routine clinical implementation alone (45). These considerations underscore that digital twin–enabled prediction frameworks should presently be regarded as evolving translational tools whose clinical value requires rigorous prospective validation before widespread adoption (45, 46).

Unlike traditional models that produce a static risk estimate at a single time point, digital twin frameworks support continuous, real-time risk assessment that adapts as new data become available (41). This alignment more closely reflects the inherently dynamic nature of cardiometabolic disease.

### 3.2 Establishing personalized reference intervals

A fundamental challenge in current clinical practice is the use of population-derived reference intervals to define “normal” biomarker values. Such one-size-fits-all thresholds assume that the same absolute value carries identical clinical significance across all individuals, regardless of age, sex, genetic background, or other intrinsic characteristics (47). This assumption overlooks the substantial interindividual variability that exists even among healthy individuals and may contribute to misclassification of risk.

To address this limitation, alternative approaches have been proposed that combine large-scale population datasets, such as the UK Biobank, with advanced statistical modeling to enable more individualized risk estimation based on genetic, demographic, and physiological characteristics (48, 49). Instead of comparing an individual's biomarker level to a fixed population percentile, the focus shifts to measuring deviation from this individualized reference (50). In this framework, what constitutes “normal” is not an absolute threshold but the degree of change relative to one's own expected trajectory. In practice, establishing personalized reference intervals requires repeated longitudinal measurements obtained under analytically stable conditions and incorporates estimates of within-individual biological variation rather than relying solely on simple trend monitoring (50, 51). This approach enables a more robust distinction between expected physiological fluctuation and clinically meaningful deviation, thereby improving the accuracy of risk assessment and supporting more individualized clinical decision-making (47, 50, 52, 53).

## 4 Toward clinical implementation: A closed-loop logic for individualized intervention

### 4.1 From risk stratification to causal inference

A key challenge in translating biomarker discoveries into clinical practice is distinguishing association from causation. Many biomarkers identified through observational studies correlate with disease outcomes but do not necessarily represent causal drivers of disease (54, 55). Relying solely on such associative markers may lead to ineffective or even misguided therapeutic strategies, particularly when selecting patients for targeted interventions (54). Importantly, predictive modelling and causal inference address complementary but fundamentally distinct analytical objectives (56, 57). A well-performing prediction model may accurately estimate an individual's future risk without identifying the biological mechanisms responsible for disease development, whereas causal inference seeks to determine whether modifying a specific biological pathway is likely to alter clinical outcomes (56, 58). Consequently, high predictive performance alone should not be interpreted as evidence of causality, particularly when prediction models are used to inform therapeutic decision-making (57, 58). Integrating predictive accuracy with causal evidence therefore represents an essential step toward biologically informed precision cardiometabolic medicine.

Mendelian randomization has emerged as a powerful approach to infer causality from large-scale observational data (54). However, future precision cardiometabolic frameworks will likely require a broader causal inference toolkit. Complementary approaches, including target-trial emulation, causal graphical models, counterfactual prediction, and methods addressing time-varying confounding, may help identify actionable intervention targets within complex longitudinal and multimodal datasets (59–62). In the context of inflammation and cardiovascular disease, for example, Mendelian randomization can clarify whether elevated inflammatory markers play a causal role in disease pathogenesis or simply accompany underlying pathological processes (63). Such causal insights are essential for guiding the appropriate use of targeted therapies, such as interleukin-6 inhibitors, ensuring that treatment is directed toward patients most likely to benefit (63). Importantly, causal hypotheses generated through computational modelling should ultimately be confirmed through prospective intervention studies before routine clinical implementation (64). Moving beyond association-based risk stratification toward causal inference thus enables a more precise and biologically grounded approach to therapeutic decision-making.

### 4.2 Dynamic monitoring and adaptive therapy

**Figure 2:**
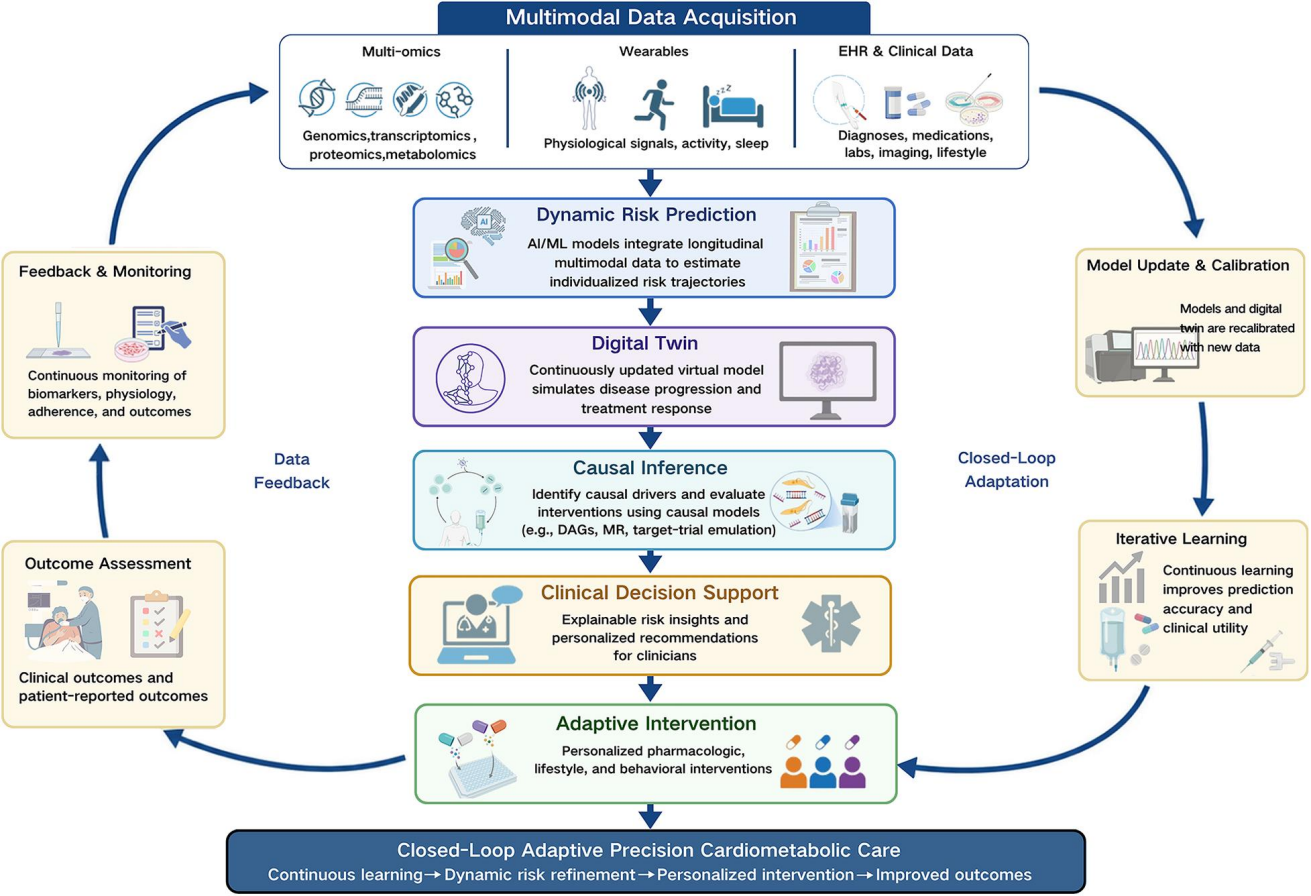
Closed-loop framework for dynamic risk prediction and adaptive cardiometabolic intervention. The proposed framework integrates longitudinal multimodal data, including omics profiles, wearable-derived physiological signals, electronic health records, and clinical assessments, into continously updated risk prediction systems. These data support individualized digital twins, causal inference analyses, and clinician-facing decision-support tools. Continous monitoring and feedback enable dynamic refinement of risk estimates and therapeutic strategies, creating a closed-loop adaptive care system that aligns clinical intervention with the evolving biological state of the individual. The figure was created using BioRender.com.

Traditional risk prediction models are typically designed as static tools that provide a one-time risk estimate based on baseline measurements (65). However, such static assessments fail to account for the dynamic nature of disease progression and treatment response (66). As patients undergo interventions, their physiological state evolves, and their risk profile may shift accordingly. Capturing these changes requires a framework that continuously integrates new information to refine risk estimates and guide ongoing treatment adjustments (65) (Figure 2).

This need has prompted a redefinition of response biomarkers—indicators that reflect an individual's biological response to an intervention and can inform subsequent therapeutic decisions (67). Although current liquid-biopsy technologies, including circulating tumor DNA and extracellular vesicles, have been developed predominantly in oncology, they provide a valuable conceptual model for dynamic biomarker surveillance in cardiometabolic medicine (68, 69). At present, however, their application to cardiometabolic diseases remains at an early stage, and disease-specific analytical validation, clinical qualification, and demonstration of incremental clinical utility will be required before routine implementation can be considered (69). Advances in liquid biopsy technologies, including circulating tumor DNA and extracellular vesicles, enable minimally invasive, repeated sampling of molecular markers that track disease dynamics (70). Wearable devices complement these data by providing continuous, real-time information on physiological parameters such as heart rate, physical activity, and sleep patterns (71). Integrating these data streams creates a closed-loop system of “treatment–feedback–readjustment” (72) (Figure 2).

To illustrate the practical application of this framework, consider a patient with type 2 diabetes and established coronary artery disease undergoing longitudinal follow-up. Multimodal data—including high-sensitivity C-reactive protein, lipid variability, continuous glucose monitoring, wearable-derived physical activity, and echocardiographic phenotypes—could be integrated into a Transformer-based prediction model to generate continuously updated individualized cardiovascular risk estimates (73, 74). Rather than serving as a static prognostic tool, such a model would support iterative risk reassessment as new clinical information becomes available, thereby facilitating timely adjustment of preventive and therapeutic strategies (74, 75). To establish clinical credibility, model performance should be evaluated not only through discrimination metrics, such as the time-dependent area under the receiver operating characteristic curve, but also through external validation, calibration, and decision-curve analysis (74). Ultimately, prospective assessment of major adverse cardiovascular events will be essential to determine whether this adaptive prediction framework translates into meaningful improvements in real-world clinical outcomes.

Within this framework, risk estimates can be continuously updated as new clinical, molecular, and physiological data become available and may be integrated into clinician-facing decision-support platforms (76, 77). Predefined risk thresholds can facilitate timely reassessment or therapeutic modification, whereas adaptive alert strategies and clinically meaningful action thresholds may help minimise unnecessary notifications (78, 79). Effective implementation will further require robust data governance, informed patient participation, and clearly defined clinical accountability (80). In our view, the ultimate value of cardiometabolic biomarkers will not lie in improving risk prediction alone. Their broader significance will depend on whether they can support timely intervention, facilitate adaptive treatment adjustment, and generate measurable improvements in patient outcomes. Consequently, future development should prioritise clinical utility and implementation feasibility alongside predictive performance.

## 5 Challenges and ethical considerations

### 5.1 Alignment of multimodal data and handling of missing values

Integrating multimodal data presents considerable technical challenges (81). Clinical records are often collected irregularly and vary in completeness across patients, whereas omics data typically consist of thousands of molecular features measured simultaneously (82, 83). This disparity leads to the well-known “curse of dimensionality,” where the number of features greatly exceeds the number of observations (81, 84). In addition, aligning data across different modalities—each with its own structure and missingness patterns—requires sophisticated preprocessing and imputation methods (85). Developing robust approaches to handle missing data without introducing bias, while preserving the biological signal within each modality, remains a key methodological priority (85).

### 5.2 Interpretability and clinical trust

Many advanced predictive models, especially those based on deep learning, function as “black boxes” (86). They can produce accurate predictions but offer no insight into the reasoning behind them. This lack of interpretability presents a major obstacle to clinical adoption. Clinicians are unlikely to trust or act on a model's recommendation if they cannot understand which factors contributed to the risk estimate (86). To address this, explainable artificial intelligence has become an essential component of models intended for clinical use (87, 88). Rather than outputting a single risk score, such models should be able to identify, for example, which specific metabolic network dysregulation is driving an increase in risk (88, 89).

Despite their promise, advanced artificial intelligence systems face several barriers prior to clinical deployment (76, 90). Model performance may deteriorate over time due to model drift, changing patient populations, evolving clinical practices, and measurement variability (91). Missing data, sensor noise, and incomplete follow-up can further compromise prediction reliability (92). Moreover, regulatory approval, medico-legal accountability, and prospective demonstration of clinical benefit remain essential prerequisites for implementation (93, 94). Consequently, dynamic prediction models should be viewed as decision-support tools requiring rigorous external validation rather than as autonomous clinical decision-makers (95).

Furthermore, the clinical value of increasingly sophisticated prediction models should not be judged solely by improvements in discrimination performance (96, 97). Comprehensive evaluation should also include reproducibility across diverse healthcare settings, robustness under external validation, calibration stability over time, and evidence that model-guided decision-making leads to meaningful improvements in patient outcomes (96–98). Such multidimensional assessment is essential to determine whether enhanced algorithmic complexity translates into genuine clinical utility and sustainable implementation in real-world precision cardiometabolic care.

### 5.3 Fairness and generalizability

Another critical concern is the risk of algorithmic bias embedded in the data used to train predictive models (99). Many large-scale biomedical databases, such as the UK Biobank, predominantly include individuals of European ancestry (99). Models developed on such datasets may not generalize well to populations with different genetic backgrounds, environmental exposures, or socioeconomic circumstances. If deployed without careful validation, these models may perpetuate or even worsen existing health disparities. Ensuring fairness requires rigorous calibration of models across diverse populations, including underrepresented racial and ethnic groups and individuals from varied socioeconomic backgrounds (100, 101). Moreover, model performance should be evaluated not only by overall predictive accuracy but also by consistency across clinically relevant subgroups (99, 100).

Beyond algorithmic bias, the implementation of precision cardiometabolic systems raises broader ethical considerations. These include governance of continuously collected health data, informed consent for longitudinal monitoring, protection of privacy in wearable- and omics-derived information, and transparency in algorithm-assisted clinical decision-making (102–104). In addition, models developed using data from large academic centres or biobank-based cohorts may not translate effectively to community, rural, or resource-limited healthcare settings (105, 106). Addressing these challenges is essential to ensure that advances in precision cardiometabolic medicine remain both equitable and broadly applicable across diverse populations.

## 6 Summary

The redefinition of cardiometabolic biomarkers reflects a fundamental epistemological shift—moving from the identification of statistical associations toward a deeper understanding of individual-level biological mechanisms. This transition moves beyond population-average frameworks to embrace a paradigm that accounts for temporal dynamics, network-based architecture, and causal structures inherent in human pathophysiology.

From our perspective, the future of cardiometabolic biomarker science should not be defined merely by the accumulation of larger datasets or increasingly complex algorithms. Rather, the central objective should be the development of biologically grounded decision systems capable of continuously integrating temporal biomarker trajectories, network-level disease mechanisms, and causal therapeutic targets. We therefore argue that the next generation of biomarkers should be evaluated not only for predictive accuracy but also for their ability to support mechanistic interpretation, individualised intervention, and real-world clinical actionability. This shift represents a transition from technology-centred innovation toward patient-centred biological decision-making and constitutes the conceptual foundation of the framework proposed in this perspective article.

To translate this vision into clinical reality, several priorities should guide future development. These include the establishment of longitudinal cardiometabolic biomarker repositories, the development of interoperable multimodal data infrastructures, the creation of externally validated and demographically diverse cohorts, and the implementation of interpretable and fairness-aware predictive models. Equally important will be the integration of clinician-facing decision-support platforms and the prospective evaluation of adaptive biomarker-guided intervention strategies. Collectively, these efforts will help bridge the gap between conceptual precision medicine frameworks and real-world cardiovascular care, ultimately enabling more individualized, equitable, and biologically informed risk assessment and intervention.

## Data Availability

The original contributions presented in the study are included in the article/Supplementary Material, further inquiries can be directed to the corresponding author.
